# Inter-rater agreement and reliability of the COSMIN (COnsensus-based Standards for the selection of health status Measurement Instruments) Checklist

**DOI:** 10.1186/1471-2288-10-82

**Published:** 2010-09-22

**Authors:** Lidwine B Mokkink, Caroline B Terwee, Elizabeth Gibbons, Paul W Stratford, Jordi Alonso, Donald L Patrick, Dirk L Knol, Lex M Bouter, Henrica CW de Vet

**Affiliations:** 1 Department of Epidemiology and Biostatistics and the EMGO Institute for Health and Care Research, VU University Medical Center, Amsterdam, The Netherlands; 2 Department of Public Health, Patient-reported Outcome Measurement Group, University of Oxford, Oxford, UK; 3 School of Rehabilitation Science and Department of Clinical Epidemiology and Biostatistics, McMaster University, Hamilton, Canada; 4 Health Services Research Unit, IMIM-Institut de Recerca Hospital del Mar, Parc de Salud Mar de Barcelona, Spain; 5 CIBER en Epidemiología y Salud Pública (CIBERESP), Barcelona, Spain; 6 Department of Health Services, University of Washington, Seattle, USA; 7 Executive Board of VU University Amsterdam, Amsterdam, The Netherlands

## Abstract

**Background:**

The COSMIN checklist is a tool for evaluating the methodological quality of studies on measurement properties of health-related patient-reported outcomes. The aim of this study is to determine the inter-rater agreement and reliability of each item score of the COSMIN checklist (n = 114).

**Methods:**

75 articles evaluating measurement properties were randomly selected from the bibliographic database compiled by the Patient-Reported Outcome Measurement Group, Oxford, UK. Raters were asked to assess the methodological quality of three articles, using the COSMIN checklist. In a one-way design, percentage agreement and intraclass kappa coefficients or quadratic-weighted kappa coefficients were calculated for each item.

**Results:**

88 raters participated. Of the 75 selected articles, 26 articles were rated by four to six participants, and 49 by two or three participants. Overall, percentage agreement was appropriate (68% was above 80% agreement), and the kappa coefficients for the COSMIN items were low (61% was below 0.40, 6% was above 0.75). Reasons for low inter-rater agreement were need for subjective judgement, and accustom to different standards, terminology and definitions.

**Conclusions:**

Results indicated that raters often choose the same response option, but that it is difficult on item level to distinguish between articles. When using the COSMIN checklist in a systematic review, we recommend getting some training and experience, completing it by two independent raters, and reaching consensus on one final rating. Instructions for using the checklist are improved.

## Background

Recently, a checklist for the evaluation of the methodological quality of studies on measurement properties of health-related patient-reported outcomes (HR-PROs) - the COSMIN checklist - was developed in an international Delphi study [1]. COSMIN is an acronym for COnsensus-based Standards for the selection of health status Measurement INstruments. This checklist can be used for the appraisal of the methodological quality of studies included in a systematic review of measurement properties of HR-PROs. It can also be used to design and report a study on measurement properties. Also, reviewers and editors could use it to identify shortcomings in studies on measurement properties, and to assess whether the methodological quality of such studies is high enough to justify publication.

The COSMIN checklist contains twelve boxes [1]. Ten boxes can be used to assess whether a study meets the standards for good methodological quality (ranging from 5-18 items). Nine of these boxes contain the standards for the measurement properties considered (internal consistency (box A), reliability (box B), measurement error (box C), content validity (box D), structural validity (box E), hypotheses testing (box F) and cross-cultural validity (box G), criterion validity (box H), and responsiveness (box I)), and one box contains standards for studies on interpretability (box J). In addition, one box (IRT box) contains requirements for articles in which Item Response Theory (IRT) methods are applied (4 items), and one box (Generalisability box) is included in the checklist that contains requirements for the generalisability of the results (8 items).

It is important to assess the quality of the COSMIN checklist itself. For example, it is important that different researchers, who use the COSMIN checklist to rate the same article, give the same ratings on each item. Therefore, the aim of this study is to determine the inter-rater agreement and reliability of each item score of the COSMIN checklist among potential users.

## Methods

Because the COSMIN checklist will be applied in the future to a variety of studies on different topics and study populations, with low and high quality, it was our goal to generalise the results of this study to a broad range of articles on measurement properties. In addition, the COSMIN checklist will be used by many researchers, using the instructions in the COSMIN manual as guidance. We were interested in the inter-rater agreement and reliability in this situation. Often, in an article only a selection of measurement properties are being evaluated. Consequently, only parts of the COSMIN checklist can be completed. We arbitrarily decided in advanced that (1) we aimed for four ratings for each item of the COSMIN checklist on the same article; (2) we aimed for each measurement property to be evaluated in at least 20 different articles. This was done to increase the representativity of studies and raters.

### Article selection

In this study we included articles that were representative of studies on measurement properties. We selected articles from the bibliographic database compiled by the Patient-Reported Outcome Measurement (PROM) Group, Oxford, UK http://phi.uhce.ox.ac.uk. The bibliography includes evaluations of PROs with information about psychometric properties and operational characteristics, and applications where for example a PRO has been used in a trial as a primary or secondary endpoint. The online PROM database comprises records downloaded from several electronic databases using a comprehensive search strategy (details available on request). The selection of articles for this study was a two-step procedure. First, of the 30,000+ included articles it was determined, based on the title, whether it concerned an article of a study on the evaluation of measurement properties of a PRO. For example, the title included terms of a specific measurement property, such as reliability, validity, or responsiveness. A total of 5137 articles were eligible. Second, from these articles, we randomly selected studies that fulfilled our inclusion criteria.

Inclusion criteria were:

• Purpose of the study was to evaluate one or more measurement properties

• Instrument under study was a HR-PRO instrument

• English language publications

Articles from any setting and any population could be included, and articles could have used Classical Test Theory (CTT) or modern test theory (i.e, Item Response Theory (IRT)) or both.

Exclusion criteria:

• Systematic reviews, case reports, letters to editors

• Studies that evaluated construct validity of two or more instruments at the same time by correlating the scores of the instruments mutually, without indicating one of instruments as the instrument of interest. In these studies, it is unclear of which instrument the construct validity is being assessed.

One of the authors (LM) selected articles until each measurement property was assessed in at least 20 articles. It appeared that we needed to select 75 articles. For each included article LM determined the relative workload for a rater to evaluate the methodological quality of the article, i.e. high, moderate, or low workload. The relative workload was based on the number of measurement properties assessed in the study, the number of instruments that were studied, the number of pages, and whether IRT was used. For example, an article in which IRT is used is considered having a high workload, and an article in which three measurement properties were evaluated in a four page paper was considered as having a low workload. We decided to ask each rater to evaluate three articles. We provided each rater with one article with a low workload, one with a moderate workload and one with a high workload.

### Selection of participants

Raters were professionals who had some experience with assessing measurement properties. This could range from having little experience to being an expert. We choose to select a heterogeneous group of raters, because this reflects best the raters who will potentially use the COSMIN checklist in the future. We invited the international panel of the COSMIN Delphi study [1] to participate in the inter-rater agreement and reliability study (n = 91), attendees of two courses on clinimetrics given in 2009 by the department of Epidemiology and Biostatistics of the VU University Medical Center (n = 72), researchers on the mailing list of the Dutch chapter of the International Society for Quality of Life Research (ISOQOL-NL) (n = 295), members of the EMGO Clinimetrics working group (n = 32), members of the PRO Methods Group of the Cochrane Collaboration (n = 79), researchers who previously showed interest in the COSMIN checklist (n = 15), colleagues of the authors, and other researchers who were likely to show interest. We also asked these people if they knew other researchers who were interested in participating.

### Procedure

Those who agreed to participate received three selected articles, together with a manual of the COSMIN checklist [2] and a data collection form to enter their scores. For each article, they were asked to follow all the COSMIN evaluation steps. Step 1: to indicate, for each measurement property, whether it was evaluated in the article ('yes/no'). The participants had to determine themselves which boxes they should complete for each of the three papers. Step 2: they were asked whether IRT was used in the article, and if so, they were asked to complete the IRT box. Step 3: they were asked to complete the relevant boxes of the COSMIN checklist. Step 4: raters were asked to complete the Generalisability box for each measurement property assessed in the article.

Instructions on how to complete the boxes were provided in the COSMIN manual [2]. Raters did not receive any additional training in completing the COSMIN checklist and were not familiar with the checklist. Items could be answered with "yes"/"no", with "yes"/"?"/"no", or with "yes"/"no"/"not applicable" ("na"). One item had four response options, i.e., "yes"/"?"/"no"/or "na".

### Statistical analyses

Each rater scored three of the 75 selected articles, and in each article a selection of the measurement properties was evaluated. Therefore, we analyzed each COSMIN item score using a one-way design.

We calculated percentage agreement for each item. This measure indicates how often raters who rated the same items on the same articles choose the same response category. We considered the highest number of similar ratings per item per article as agreement, and the other ratings as non-agreement. For example, if five raters rated the same item for the same article, and three of the raters rated 'yes', and two rated 'no', we considered three ratings as agreement. Percentage agreement was calculated by the number of ratings with agreement on all articles, divided by the total number of ratings on all articles for which that measurement property was assessed. A percentage agreement > 80% was considered appropriate (arbitrarily chosen).

In addition, we calculated the reliability of the items using kappa coefficients. This is a measure that indicates how well articles can be distinguished from each other based on the given COSMIN item score. Dichotomous items were analysed using intraclass kappa coefficients [3]; the scoring was yes = 1 and no = 0.

$$Intraclass\;Kappa_{COSMINitem}=\frac{\sigma_{article}^{2}}{\sigma_{article}^{2}+\sigma_{error}^{2}},$$

where σ^2^_article _denotes the variance due to systematic differences between the articles for which the item was scored, and σ^2^_error _denotes the random error.

Ordinal items were analyzed with weighted kappa coefficients using quadratic weights; the scoring was 'yes' = 1, '?' = 2, and 'no' = 3. (Note that the scorings order in the COSMIN checklist is yes/no/?). These measures are numerically the same as intraclass correlation coefficients (ICCs) obtained from analysis of variance (ANOVA) [4-6].

Twenty-two items could be answered with "na", which makes the scale of these items a multi-categorical nominal scale. For these items, we calculated for each item kappa's after all possible dichotomizations. For example, item A9 has three response options, i.e. 'yes', 'no', and 'na'. This item has three times been dichotomized, i.e. into yes = 1 and not yes = 0 (dummy variable 1), into no = 1 and not no = 0 (dummy variable 2), and into na = 1 and not na = 0 (dummy variable 3). Next, the components for the intraclass kappa were calculated, and a summary intraclass (SI) kappa was calculated using formula [3]

$$SI\;Kappa_{COSMIN\text{​}item}=\frac{\sum_{i}\sigma_{article}^{2}(i)}{\sum_{i}\sigma_{article}^{2}(i)+\sum_{i}\sigma_{error}^{2}(i)}.$$

The numerator reflects the variance due to the article, and the denominator reflects the total variance. In case a variance component was negative, we set the variance at zero.

Since we do not calculate overall scores per box, we only calculated kappa coefficients per COSMIN item. We considered a kappa for each item below 0.40 as poor, between 0.40 and 0.75 as moderate to good, and above 0.75 as excellent [6].

Reliability measures such as kappa are dependent on the distribution of the data (σ^2^_article_). Vach showed that reliability measures are low when data are skewed [7]. We considered a distribution of scores as skewed when more than 75% of the raters who responded to an item used the same response category. Percentage agreement is not dependent on the distribution of the data.

In our analysis we combined scores of the items on the Generalisability box for all measurement properties, so that we calculated percentage agreement and kappa coefficients only once for each of the items from this box, and not separately for each measurement property.

## Results

A total of 154 raters agreed to participate in this study. We received the ratings from 88 (57%) of the participants. The responders came from the Netherlands (58%), Canada (10%), UK (7%), Australia or New Zealand (6%), Europe without Netherlands and UK (15%), other (5%). The mean number of years experience in research was 12 years (SD = 8.7), and 9 years (SD = 7.1) experience in research related to measurement properties.

Of the 75 selected articles, 8 articles were rated by six participants, 7 articles were rated by five participants, 11 by four participants, 38 by three participants, and 11 by two participants. The percentage missing items per box were 7% for box A Internal Consistency (11 item), 5% for box B Reliability (14 items), 1% box D Content Validity (5 items), 11% box E Structural Validity (7 items), 7% box F Hypotheses Testing (10 items), 5% box G Cross-cultural Validity (15 items), 5% box H Criterion Validity (7 items), 18% box I Responsiveness (18 items), 3% box J Interpretability (9 items), and 1% for the Generalisability box (8 items).

Items of the IRT box had 26 ratings for 13 articles; for 6 articles this box was completed by one rater, for two articles by two raters, for four articles by three raters, and for one article by four raters. The box C Measurement error had 17 ratings for 14 articles; for twelve articles this box was completed by one rater, for one article by two raters, and one article by three raters. The results of these items are not shown, because percentage agreement and kappa coefficients based on such small numbers are unreliable. For the property measurement error, however, we have some information because 10 of the 11 items from this box (i.e. all items on design requirements) were exactly the same items as the items about design requirements from box B Reliability (i.e. items B1 to B10).

Table 1 shows the inter-rater agreement and reliability of the questions regarding whether the property was evaluated in an article (step 1 of the COSMIN checklist). Note that these scores are not summary scores of the overall methodological quality of the property. All properties had high percentage agreement (range from 84% to 96%).Two of the ten properties, i.e. Reliability and Responsiveness, had an excellent kappa coefficient, i.e. above 0.75. Three properties had moderate to good kappa coefficients and five had poor kappa coefficients.

**Table 1:** Inter-rater agreement (percentage agreement) and reliability (kappa coefficients) on whether the property was evaluated in an article (COSMIN step 1)

	percentage agreement	Intraclass kappa^a^
Internal consistency	**94**	0.66
Reliability	**94**	**0.77**
Measurement error	**94**	0.02^b^
Content validity	**84**	0.29
Structural validity	**86**	0.48
Hypotheses testing	**87**	0.29
Cross-cultural validity	**95**	0.66^b^
Criterion validity	**93**	0.23^b^
Responsiveness	**96**	**0.81**
Interpretability	**86**	0.02^b^

^a ^number of ratings on the 75 articles = 263; *^b ^*items with low dispersal i.e. more than 75% of the raters who responded to an item rated the same response category; printed in bold indicates kappa > 0.70 or % agreement >80%

In Table 2 we describe percentages agreement, and kappa coefficients for each item of the COSMIN boxes A to J (step 3). Fifty-nine items (61%) of the 96 items in Table 2 had a percentage agreement above 80%. Thirty items (31%) had a percentage agreement between 70% and 80%, and seven items (7%) between 60% and 70%. Of the 96 items, five (5%) had an excellent kappa coefficient, thirty (31%) had a moderate to good kappa coefficient, and 61 items (64%) had a poor kappa coefficient (including the 15 items of which we set negative variance components to 0). Sample sizes for percentage agreement and kappa coefficients per item were slightly different, due to articles that were scored only once by one rater. When calculating percentage agreement, these articles could not be taken into account.

**Table 2:** Inter-rater agreement (percentage agreement) and reliability (kappa coefficients) of the items from the COSMIN checklist (COSMIN step 3)

Item nr	Item	N (minus articles with 1 rating)^a^	% agreement	N	Kappa
**Box A Internal consistency (n = 195)^b^**					
A1	Does the scale consist of effect indicators, i.e. is it based on a reflective model?	185	**82**	193	0.06
Design requirements					
A2^c ^	Was the percentage of missing items given?	183	**87**	190	0.48
A3^c^	Was there a description of how missing items were handled?	180	**90**	187	0.54
A4	Was the sample size included in the internal consistency analysis adequate?	177	**87**	185	0.06^d^
A5^c^	Was the unidimensionality of the scale checked? i.e. was factor analysis or IRT model applied?	180	**92**	187	0.69
A6	Was the sample size included in the unidimensionality analysis adequate?	166	79	178	0.27
A7	Was an internal consistency statistic calculated for each (unidimensional) (sub)scale separately?	179	**85**	187	0.31^d^
A8^c^	Were there any important flaws in the design or methods of the study?	174	**86**	179	0.22^d^
Statistical methods					
A9	for Classical Test Theory (CTT): Was Cronbach's alpha calculated?	179	**93**	187	0.27^d,e^
A10	for dichotomous scores: Was Cronbach's alpha or KR-20 calculated?	151	**91**	165	0.17^d,e^
A11	for IRT: Was a goodness of fit statistic at a global level calculated? e.g. χ^2^, reliability coefficient of estimated latent trait value (index of (subject or item) separation)	154	**93**	167	0.46^d,e^
					
**Box B. Reliability (n = 141)^b^**					
Design requirements					
B1^c^	Was the percentage of missing items given?	129	**87**	140	0.39
B2^c^	Was there a description of how missing items were handled?	125	**91**	137	0.43^d^
B3	Was the sample size included in the analysis adequate?	127	77	139	0.35
B4^c^	Were at least two measurements available?	129	**98**	140	**0.72**^d^
B5	Were the administrations independent?	129	73	139	0.18
B6^c^	Was the time interval stated?	125	**94**	136	0.50^d^
B7	Were patients stable in the interim period on the construct to be measured?	126	75	138	0.24
B8	Was the time interval appropriate?	125	**84**	137	0.45
B9	Were the test conditions similar for both measurements? e.g. type of administration, environment, instructions	127	**83**	138	0.30
B10^c^	Were there any important flaws in the design or methods of the study?	117	77	129	0.08
Statistical methods					
B11	for continuous scores: Was an intraclass correlation coefficient (ICC) calculated?	119	**86**	133	0.59^e ^
B12	for dichotomous/nominal/ordinal scores: Was kappa calculated?	111	**81**	127	0.32^e^
B13	for ordinal scores: Was a weighted kappa calculated?	111	**83**	127	0.42^e^
B14	for ordinal scores: Was the weighting scheme described? e.g. linear, quadratic	108	**81**	124	0.35^e^
					
**Box D. Content validity (n = 83)^b^**					
Design requirements					
D1	Was there an assessment of whether all items refer to relevant aspects of the construct to be measured?	62	79	83	0.33
D2	Was there an assessment of whether all items are relevant for the study population? (e.g. age, gender, disease characteristics, country, setting)	62	76	83	0.46
D3	Was there an assessment of whether all items are relevant for the purpose of the measurement instrument? (discriminative, evaluative, and/or predictive)	62	66	83	0.21
D4	Was there an assessment of whether all items together comprehensively reflect the construct to be measured?	62	66	83	0.15
D5^c^	Were there any important flaws in the design or methods of the study?	58	76	78	0.13
					
**Box E. Structural validity (n = 118)^b^**					
E1	Does the scale consist of effect indicators, i.e. is it based on a reflective model?	99	78	116	0^f^
Design requirements					
E2^c^	Was the percentage of missing items given?	95	**87**	110	0.41
E3^c^	Was there a description of how missing items were handled?	93	**91**	109	0.55
E4	Was the sample size included in the analysis adequate?	94	**87**	109	0.56^d^
E5^c^	Were there any important flaws in the design or methods of the study?	89	**84**	103	0.27
Statistical methods					
E6	for CTT: Was exploratory or confirmatory factor analysis performed?	92	**90**	106	0.51^d,e^
E7	for IRT: Were IRT tests for determining the (uni-) dimensionality of the items performed?	62	**87**	80	0.39^e,f^
					
**Box F. Hypotheses testing (n = 170)^b^**					
Design requirements					
F1^c^	Was the percentage of missing items given?	158	**87**	168	0.41
F2^c^	Was there a description of how missing items were handled?	159	**92**	169	0.60^d^
F3	Was the sample size included in the analysis adequate?	157	**84**	167	0.12^d^
F4	Were hypotheses regarding correlations or mean differences formulated a priori (i.e. before data collection)?	158	74	168	0.42
F5	Was the expected direction of correlations or mean differences included in the hypotheses?	159	75	169	0.26^e^
F6	Was the expected absolute or relative magnitude of correlations or mean differences included in the hypotheses?	159	**82**	168	0.29^e^
F7^c^	for convergent validity: Was an adequate description provided of the comparator instrument(s)?	125	**83**	136	0.30
F8^c^	for convergent validity: Were the measurement properties of the comparator instrument(s) adequately described?	124	**81**	135	0.35
F9^c^	Were there any important flaws in the design or methods of the study?	131	**81**	145	0.17
Statistical methods					
F10	Were design and statistical methods adequate for the hypotheses to be tested?	150	78	161	0.00^d,e,f^
					
**Box G. Cross-cultural validity (n = 33)^b^**					
Design requirements					
G1^c^	Was the percentage of missing items given?	25	**88**	32	0.52
G2^c^	Was there a description of how missing items were handled?	22	**82**	30	0.32
G3	Was the sample size included in the analysis adequate?	26	**81**	33	0.23
G4^c^	Were both the original language in which the HR-PRO instrument was developed, and the language in which the HR-PRO instrument was translated described?	28	**89**	33	0.34^d^
G5^c^	Was the expertise of the people involved in the translation process adequately described? e.g. expertise in the disease(s) involved, expertise in the construct to be measured, expertise in both languages	28	**86**	33	0.46
G6	Did the translators work independently from each other?	28	**89**	33	0.61
G7	Were items translated forward and backward?	28	**100**	33	**1.00**
G8^c^	Was there an adequate description of how differences between the original and translated versions were resolved?	28	**86**	33	0.50
G9^c^	Was the translation reviewed by a committee (e.g. original developers)?	25	**88**	31	0.56
G10^c^	Was the HR-PRO instrument pre-tested (e.g. cognitive interviews) to check interpretation, cultural relevance of the translation, and ease of comprehension?	21	**90**	29	0.61
G11^c^	Was the sample used in the pre-test adequately described?	28	79	32	0^f^
G12	Were the samples similar for all characteristics except language and/or cultural background?	26	**81**	31	0.41
G13^c^	Were there any important flaws in the design or methods of the study?	26	**85**	31	0.42
Statistical methods					
G14	for CTT: Was confirmatory factor analysis performed?	27	74	32	0.03^e,f^
G15	for IRT: Was differential item function (DIF) between language groups assessed?	13	77	23	0.28^e,f^
					
**Box H. Criterion validity (n = 57)^b^**					
Design requirements					
H1^c^	Was the percentage of missing items given?	35	**91**	56	0.59^d^
H2^c^	Was there a description of how missing items were handled?	35	**97**	56	**0.79**^d^
H3	Was the sample size included in the analysis adequate?	35	69	54	0.06
H4	Can the criterion used or employed be considered as a reasonable 'gold standard'?	37	62	57	0^f^
H5^c^	Were there any important flaws in the design or methods of the study?	33	79	54	0.10
Statistical methods					
H6	for continuous scores: Were correlations, or the area under the receiver operating curve calculated?	37	78	56	0.16^e ^
H7	for dichotomous scores: Were sensitivity and specificity determined?	29	**83**	47	0.28^e,f^
					
**Box I. Responsiviness (n = 79)^b^**					
Design requirements					
I1^c^	Was the percentage of missing items given?	71	**82**	76	0.14^d^
I2^c^	Was there a description of how missing items were handled?	73	**92**	77	0.36^d^
I3	Was the sample size included in the analysis adequate?	72	72	76	0.40
I4^c^	Was a longitudinal design with at least two measurement used?	73	**100**	78	**1.00**^d^
I5^c^	Was the time interval stated?	73	**89**	78	0.25^d^
I6^c^	If anything occurred in the interim period (e.g. intervention, other relevant events), was it adequately described?	72	78	75	0.17
I7^c^	Was a proportion of the patients changed (i.e. improvement or deterioration)?	70	**97**	73	0.32^d^
Design requirements for hypotheses testing					
For constructs for which a gold standard was not available					
I8	Were hypotheses about changes in scores formulated a priori (i.e. before data collection)?	65	69	72	0.35
I9	Was the expected direction of correlations or mean differences of the change scores of HR-PRO instruments included in these hypotheses?	60	78	65	0.19^e^
I10	Were the expected absolute or relative magnitude of correlations or mean differences of the change scores of HR-PRO instruments included in these hypotheses?	61	**90**	66	0.05^d,e^
I11^c^	Was an adequate description provided of the comparator instrument(s)?	56	70	63	0^f^
I12^c^	Were the measurement properties of the comparator instrument(s) adequately described?	56	**80**	63	0.06
I13^c^	Were there any important flaws in the design or methods of the study?	63	71	68	0.03
Statistical methods					
I14	Were design and statistical methods adequate for the hypotheses to be tested?	63	73	67	0.21^e,f^
Design requirements for comparison to a gold standard					
For constructs for which a gold standards was available:					
I15	Can the criterion for change be considered as a reasonable 'gold standard'?	21	67	28	0^f^
I16^c^	Were there any important flaws in the design or methods of the study?	12	67	21	0^f^
Statistical methods					
I17	for continuous scores: Were correlations between change scores, or the area under the Receiver Operator Curve (ROC) curve calculated?	28	79	39	0.47^e,f^
I18	for dichotomous scales: Were sensitivity and specificity (changed versus not changed) determined?	28	79	37	0.15^e^
					
**Box J. Interpretability (n = 42)^b^**					
J1^c^	Was the percentage of missing items given?	22	**95**	41	**0.80**
J2^c^	Was there a description of how missing items were handled?	21	76	41	0.19
J3	Was the sample size included in the analysis adequate?	23	74	41	0^f^
J4^c^	Was the distribution of the (total) scores in the study sample described?	23	74	41	0.08
J5^c^	Was the percentage of the respondents who had the lowest possible (total) score described?	20	**95**	40	**0.84**
J6^c^	Was the percentage of the respondents who had the highest possible (total) score described?	21	**90**	41	**0.70**
J7^c^	Were scores and change scores (i.e. means and SD) presented for relevant (sub) groups? e.g. for normative groups, subgroups of patients, or the general population	21	76	41	0.05
J8^c^	Was the minimal important change (MIC) or the minimal important difference (MID) determined?	19	**89**	40	0.26^d^
J9^c^	Were there any important flaws in the design or methods of the study?	21	71	41	0^f^

^a ^When calculating percentage agreement, articles that were only scored once on the particular item were not taken into account;^b ^number of times a box was evaluated;^c ^dichotomous item;^d ^Items with low dispersal i.e. more than 75% of the raters who responded to an item rated the same response category;^e ^Combined kappa coefficient calculated because of nominal response scale in a one-way design;^f ^Negative variance component in the calculation of kappa was set at 0;^g ^sample sizes of Generalisability box are much higher that other items, because scores of the items on the Generalisability box for all measurement properties were combined; printed in bold indicates Kappa > 0.70 or % agreement >80%.

In Table 3 percentages agreement and kappa coefficients are given for the eight items from the Generalisability box (step 4). We combined scores of the items on the Generalisability box for all measurement properties. Therefore, the sample sizes are much higher. All items in Table 3 had a percentage agreement above 80%. None of the items had an excellent kappa coefficient. Four items had a moderate to good kappa coefficient, and four items had a poor kappa coefficient.

**Table 3:** Inter-rater agreement (percentage agreement) and reliability (kappa coefficients) of the items from the COSMIN checklist (COSMIN step 4)

Item nr	Item	N (minus articles with 1 rating)^a^	% agreement	N	Kappa
**Generalisability Box (n = 866)^b^**		^c^			
Was the sample in which the HR-PRO instruments was evaluated adequately described? In terms of:					
1^d^	median or mean age (with standard deviation or range)?	733	**86**	865	0.36
2^d^	distribution of sex?	735	**88**	863	0.38^e^
3	important disease characteristics (e.g. severity, status, duration) and description of treatment?	746	**80**	862	0.39^f^
4^d^	setting(s) in which the study was conducted? e.g. general population, primary care or hospital/rehabilitation care	735	**89**	863	0.30^e^
5^d^	countries in which the study was conducted?	733	**90**	861	0.40^e^
6^d^	language in which the HR-PRO instrument was evaluated?	733	**86**	861	0.41^e^
7^d^	Was the method used to select patients adequately described? e.g. convenience, consecutive, or random	729	**81**	857	0.40
8	Was the percentage of missing responses (response rate) acceptable?	724	**82**	849	0.48

^a ^When calculating percentage agreement, articles that were only scored once on the particular item were not taken into account;^b ^number of times a box was evaluated;^c ^sample sizes of Generalisability box are much higher that other items, because scores of the items on the Generalisability box for all measurement properties were combined;^d ^dichotomous item;^e ^Items with low dispersal i.e. more than 75% of the raters who responded to an item rated the same response category;^f ^Combined kappa coefficient calculated because of nominal response scale in a one-way design; printed in bold indicates Kappa > 0.70 or % agreement >80%.

We observed two issues. Firstly, thirty-two of the 114 items (Table 1, 2 and 3; 28%) showed hardly any dispersal, i.e. more than 75% of the raters who responded to the item rated the same response category. When data are skewed, the between article variance, i.e. σ^2^_article_, is low, and thus the kappa will be low. Secondly, in Table 2 it can be seen that twenty-nine items (28%) had a sample size below 50 for the calculation of kappa coefficients, of which four were below 30 (4%). For the calculation of percentage agreement thirty-five items (34%) had a sample size of below 50, of which twenty-nine (28%) was below 30. These percentage agreement and kappa coefficients based on such small numbers should be interpreted with caution.

## Discussion

In this study we investigated the inter-rater agreement and reliability of the item scores on the COSMIN checklist. Overall, the percentages agreement were high, indicating that raters often choose the same response option. The kappa coefficients were low, indicating that it is difficult to distinguish on item level between articles. We will start the discussion with reasons for low kappa coefficients, and for low percentages of agreement.

Although the term *inter-rater agreement *does not appear in the COSMIN taxonomy [8], we used it in this study. For measurement instruments that have continuous scores the measurement error can be investigated. However, instruments with a nominal or ordinal score do not have a unit of measurement, and consequently, measurement error can not be calculated. Because we were interested in whether the ratings were similar, we present the percentage agreement of all nominal and ordinal items.

### Reasons for low kappa coefficients

Kappa coefficients for 70 of the 114 items were poor. This is partly due to a skewed distribution of the item scores. Low dispersal rates strongly influence the kappa, because if the variance between articles is low, the error variance is large in relation to the article variance. For example, item I5 of the box Responsiveness (i.e. was the time interval stated) had a kappa of 0.25; 65 times raters scored "yes" (83%), and 13 times they scored "no" (17%).

### Reasons for low inter-rater agreement between raters

Percentage agreement was below 80% in 37 of the 114 items. For many items of the COSMIN checklist a subjective judgement is needed. For example, in each box the item *'were there are any important flaws in the design or the methods of the study' *was included (e.g., B10, I13, I16 and J9). To answer this question, the rater should judge this based on his own experience and knowledge. Therefore, some kind of subjective evaluation is involved. Some other items might be rather difficult to score, because the information needed to answer the item is not reported in the article. For example, information to be able to respond on the item *'were the administrations independent' *(B5) is often not reported. Although raters should score '?' in this case, raters are likely to guess, or skip these items. This influences the kappa coefficients and the percentage agreement.

Furthermore, the COSMIN checklist contains consensus-based standards that may deviate from how persons are used to evaluate measurement properties or a person may disagree on a particular item. Consequently, a rater may score an item differently than recommended in the COSMIN manual. For example, many people consider effect sizes as appropriate measures for responsiveness. Within the COSMIN Delphi study, we decided to consider this as inappropriate [9]. We believe that only when clear hypotheses are formulated about the expected magnitude of the effect sizes (ES) it is appropriate as an indicator of responsiveness (I14). Another example is the issue about the gold standard. The COSMIN panel considered a commonly used measurement instrument, such as the SF-36, not as a reasonable gold standard. However, raters may disagree with this, and rate the item '*can the criterion (for change) be considered as a reasonable gold standards' *(H4 and I15) as 'yes' while according to the COSMIN manual this item should be scored with 'no'. Consequently, the kappa coefficient and the percentage agreement will be low.

Last, the distinction between rating the methodological quality of the study and rating the quality of the instrument that is evaluated in the study may be difficult, especially for content validity. Therefore, the items on content validity are difficult to score. All items of box D of content validity had low kappa coefficients and percentage agreement. They ask whether the article under study appropriately *investigated *whether the items were relevant and comprehensive. This refers to the methodological quality of a study. For example, an appropriate method to investigate the content validity of a HR-PRO is involving patients from the target population, by asking them about the relevance and comprehensiveness of the items. These COSMIN items do not ask whether the items of the PRO under study *are *relevant and comprehensive, which refers to the quality of an instrument. Raters may have been confused about this distinction.

### Strength and weaknesses of the study

We are confident that raters who have participated in this study are representative for the future users of the COSMIN Checklist, since the number of years of experiences in research varied widely. We used a wide range of articles that are likely to be a representative sample of articles on measurement properties. The distribution of many articles over many raters (no pairs, no ordering) enhances generalisability of our results and leads to conservative estimates. Also, we did not intervene beyond the delivery of the checklist and the instructions manual. In all, the study should be seen as a very similar to the usual conditions of its use.

It was our aim to randomly select equal numbers of studies on each measurement property. However, studies on internal consistency and hypotheses testing are more common than studies on measurement error and interpretability. Studies that are based on CTT are more common than studies that apply IRT methods. Consequently, these less common measurement properties were less often selected for this study. This prevented analysis of the items on measurement error and on IRT analysis.

In addition, it was our aim to include a representative sample of potential users of the COSMIN checklist. As expected, the years of experience of the participants in this study both in research in general and in research in measurement instruments differed widely. Although more than half of the raters came from the Netherlands, we do not expect that the country of origin will have a major influence on the results.

In this study it was not feasible to train the raters because we expected that this would dramatically decrease the response rate. However, we recommend getting some experience in completing the COSMIN checklist before conducting a systematic review. In the future, when more raters are trained in completing the checklist, a reliability study among trained raters could be performed.

Due to the incomplete study design (i.e. not all raters scored all articles, and in an article not all measurement properties are evaluated) we had a one-way design. Therefore, the variance due to raters could not be distinguished from the error variance. Other optional designs would be asking a few raters to evaluate many articles, or asking many raters to evaluate the same few articles. Both designs were considered poor. In the first case, it is likely that we would not find participants, due to the large amount of work each rater had to do. We felt that we as authors of the COSMIN checklist should not be these raters, because of our involvement in the development of the checklist. The second design is considered poor because we would have to include a few articles in which all measurement properties were evaluated. It is very likely that these articles do not exist, and if such an article is published, it is very likely that it is not a good representation of studies on measurement properties.

### Recommendations for improvement of the inter-rater agreement and reliability of the COSMIN checklist

Firstly, based on the results of this study, and feedback we received from raters, we improved the wording and grammar of a few items and we adapted the instructions in the manual. This might improve the agreement on the COSMIN item scores. Secondly, the COSMIN checklist is not a ready-made checklist, in a sense that the user can instantly complete all items. We recommend that researchers who use the COSMIN checklist, for example in a systematic review, agree beforehand on how to handle items that need a subjective judgement, and how to deal with lack of reporting in the original article. For example, based on the topic of the review, they should agree on what they consider an appropriate time interval for reliability (B8), on an adequate description for the comparator instrument(s) (F7 and I11), or on an acceptable percentage of missing responses (item 8 of the Generalisability box). This may also increase the inter-rater agreement. Thirdly, some experience in completing the checklist before conducting a systematic review is also likely to increase the inter-rater agreement of the COSMIN checklist. Therefore, we are developing a training set of articles (to be published on our website), explaining how these articles should be evaluated using the COSMIN checklist. Fourthly, we strongly recommend using the taxonomy and terminology of the COSMIN checklist. For example, if authors compare their PRO to a commonly used PRO such as the SF-36, and they refer to this as criterion validity, we recommend considering this an evaluation of hypotheses testing which is an aspect of construct validity, and complete box F. Fifthly, when using the checklist in a systematic review of HR-PROs, we recommend to complete the checklist by at least two independent raters, and to reach consensus on one final rating. In this study we used the ratings of single raters to determine the inter-rater agreement of the checklist, because a design with consensus scores of two raters was not feasible. We recommend evaluating the inter-rater agreement of the consensus scores of couples of raters in a future study, when more raters are trained.

Note that in this study, we investigated the inter-rater agreement and reliability on item level. Results showed that it is difficult to distinguish articles on item level. When using the COSMIN checklist in a systematic review on measurement properties, an overall score per box is useful to decide whether the methodological quality can be considered as good. For such a score, the reliability might be better.

### Reliability of other checklists

We found three studies in which the inter-rater agreement and reliability of a similar kind of checklist was investigated.

In one study the reliability of a 39 item appraisal tool to evaluate PRO instruments (EMPRO) [10] was investigated. In this study five panels (in which three or four raters participated) each assessed the quality of the Spanish version of one well-known and widely used PRO instrument. Intraclass correlation coefficients (two-way model, absolute agreement) were calculated both for the overall assessment of the quality of the score. High ICCs were found (all above 0.75) [10]. COSMIN and EMPRO both focus on PROs. However, with the COSMIN checklist it is not yet possible to calculate an overall score per box or an overall score about the quality of all measurement properties together. In addition, EMPRO assesses the overall quality of a measurement instrument, while COSMIN assesses the methodological quality of studies on measurement properties.

In two other studies two independent raters scored a number of articles using either STAndards for the Reporting of Diagnostic accuracy studies (STARD) [11] or Nelson-Moberg Expanded CONSORT Instrument (NMECI) [12]. Both studies reported percentage agreement and kappa coefficients. In the study by Smidt et al. [11] they found percentage agreement between 63% and 100%, and kappa coefficients between -0.032 and 1.00. About the same percentage of items as in COSMIN (61% of the STARD items) showed high percentage agreement (i.e. above 80%). However, more items had higher kappa coefficients, i.e. 23% of the STARD items showed excellent kappa coefficients (i.e above 0.70). In the study by Moberg-Mogren & Nelson [12], 77% of the CONSORT items showed high ICC (i.e. above 0.70), and 57% of the NMECI items showed high kappa coefficients (i.e. above 0.70). Of the NMECI items, 29 of the 176 kappa coefficients were below 0.40. For these items they also showed percentage agreement, ranging between 43% and 93%. CONSORT and NMECI items had higher values for reliability than the COSMIN items.

## Conclusion

The inter-rater agreement of the COSMIN items was adequate, i.e. raters mostly rated the items of the COSMIN checklist quite the same. The inter-rater reliability of the COSMIN items was poor for many items; it was difficult to distinguish between articles based on item level. Some disagreements between raters are likely to be influenced by a subjective judgement needed to answer an item. Therefore, we recommend making decisions in advance about how to score these issues. The inter-rater agreement on other items may have improved after this study since we have tried to improve the instructions in the manual on some issues, based on the feedback of raters. When using the COSMIN checklist it is important to read the manual carefully, and get some training and experience in completing the checklist.

## Competing interests

The authors except for E. Gibbons were the developers of the COSMIN checklist.

## Authors' contributions

LB, CT and HdV secured funding for the study. CT, HdV, LB, DK, DP, JA, PS, and EG conceived the idea for the study. EG prepared the database and LM selected the articles. All authors invited potential raters. LM coordinated the study and managed the data. LM, CT, DK and HdV interpreted the data. CT, EG, DP, JA, PS, DK, LB and HdV supervised the study. LM wrote the manuscript with input from all the authors. All authors read and approved the final version of the report.

## Pre-publication history

The pre-publication history for this paper can be accessed here:

http://www.biomedcentral.com/1471-2288/10/82/prepub

## References

[B1] Mokkink LB Terwee CB Patrick DL Alonso J Stratford PW Knol DL Bouter LM De Vet HCW The COSMIN checklist for assessing the methodological quality of studies on measurement properties of health status measurement instruments: an international Delphi study Qual Life Res 2010 19 539 549 10.1007/s11136-010-9606-8 20169472 PMC2852520

[B2] Mokkink LB Terwee CB Patrick DL Alonso J Stratford PW Knol DL Bouter LM De Vet HCW The COSMIN checklist manual http://www.cosmin.nl

[B3] Landis JR Koch GG A one-way components of variance model for categorical data Biometrics 1977 33 671 679 10.2307/2529465

[B4] Kraemer HC Periyakoil VS Noda A Tutorial in biostatistics. Kappa coefficients in medical research Stat Med 2002 21 2109 2129 10.1002/sim.1180 12111890

[B5] Lin L Hedayat AS Wu W A unified approach for assessing agreement for continuous and categorical data J Biopharm Stat 2007 17 629 652 10.1080/10543400701376498 17613645

[B6] Fleiss JL Statistical methods for rates and proportions 1981 New York: John Wiley & Sons

[B7] Vach W The dependence of Cohen's kappa on the prevalence does not matter J Clin Epidemiol 2005 58 655 661 10.1016/j.jclinepi.2004.02.021 15939215

[B8] Mokkink LB Terwee CB Patrick DL Alonso J Stratford PW Knol DL Bouter LM de Vet HC The COSMIN study reached international consensus on taxonomy, terminology, and definitions of measurement properties for health-related patient-reported outcomes J Clin Epidemiol 2010 63 737 745 10.1016/j.jclinepi.2010.02.006 20494804

[B9] Mokkink LB Terwee CB Knol DL Stratford PW Alonso J Patrick DL Bouter LM De Vet HCW The COSMIN checklist for evaluating the methodological quality of studies on measurement properties: A clarification of its content BMC Med Res Methodol 2010 10 22 10.1186/1471-2288-10-22 20298572 PMC2848183

[B10] Valderas JM Ferrer M Mendivil J Garin O Rajmil L Herdman M Alonso J Development of EMPRO: A tool for the standardized assessment of patient-reported outcome measures Value Health 2008 11 700 708 10.1111/j.1524-4733.2007.00309.x 18194398

[B11] Smidt N Rutjes AW Van der Windt DA Ostelo RW Bossuyt PM Reitsma JB Bouter LM De Vet HCW Reproducibility of the STARD checklist: an instrument to assess the quality of reporting of diagnostic accuracy studies BMC Med Res Methodol 2006 6 12 10.1186/1471-2288-6-12 16539705 PMC1522016

[B12] Moberg-Mogren E Nelson DL Research concepts in clinical scholarship: Evaluating the quality of reporting occupational therapy randomized controlled trials by expanding the CONSORT criteria Am J Occup Ther 2006 60 226 235 16596926 10.5014/ajot.60.2.226

